# Silver Release Diffusion Modeling and Biological Performance of Poly(Vinyl Alcohol)/Alginate Hydrogels with Embedded Silver Nanoparticles

**DOI:** 10.3390/molecules31152702

**Published:** 2026-08-03

**Authors:** Vesna Mišković-Stanković, Ana Janković, Aleksandra Perić Grujić, Maja Vukašinović Sekulić, Vesna Kojić, Marija Djošić, Milena Stevanović, Teodor M. Atanackovic

**Affiliations:** 1 Faculty of Ecology and Environmental Protection, University Union—Nikola Tesla, Cara Dusana 62-64, 11000 Belgrade, Serbia; 2 Innovation Center of the Faculty of Technology and Metallurgy, University of Belgrade, Karnegijeva 4, 11000 Belgrade, Serbia; ajankovic@tmf.bg.ac.rs (A.J.); mstevanovic@tmf.bg.ac.rs (M.S.); 3 Faculty of Technology and Metallurgy, University of Belgrade, Karnegijeva 4, 11000 Belgrade, Serbia; alexp@tmf.bg.ac.rs (A.P.G.); vukasinovic@tmf.bg.ac.rs (M.V.S.); 4 Oncology Institute of Vojvodina, University of Novi Sad, Put Dr Goldmana 4, 21204 Sremska Kamenica, Serbia; vesna.kojic@outlook.com; 5 Institute for Technology of Nuclear and Other Mineral Raw Materials, Bulevar Franš d’Eperea 86, 11000 Belgrade, Serbia; mdjosic@yahoo.com; 6 Serbian Academy of Sciences and Arts, 11000 Belgrade, Serbia; atanackovic@uns.ac.rs

**Keywords:** poly(vinyl alcohol), alginate, silver release, diffusion, modelling, cytotoxicity, antibacterial activity

## Abstract

Silver nanoparticles have been electrochemically embedded in poly(vinyl alcohol)/alginate hydrogels, producing composites with antibacterial activity and controlled silver release. UV–visible and FTIR spectroscopy confirmed the incorporation of silver nanoparticles and established bonds in composite hydrogels. The silver nanoparticle-loaded hydrogels exhibited strong antibacterial activity, achieving complete reduction of *Staphylococcus aureus* TL and *Escherichia coli* ATCC 25922 cells after only 1 h of exposure, while the MTT assay confirmed their biocompatibility, with cell viability above 80% for both L929 and MRC-5 cell lines. Silver release was measured in phosphate buffer at 37 °C using atomic absorption spectroscopy (AAS). Release kinetics was analyzed using our two-compartmental model with a general fractional derivative and compared with established approaches in diffusion modeling. The general fractional derivative (GFD) model provided the closest fit to the experimental silver release data, exhibiting the lowest fitting error values (R = 0.0014 for silver/PVA/0.5 wt% alginate and R = 0.0051 for silver/PVA/1.0 wt% alginate) and enabling the accurate determination of the silver diffusion coefficient over the entire release period. These findings demonstrate that silver/poly(vinyl alcohol)/alginate hydrogels combine biocompatibility, antibacterial efficacy, and predictable silver release, highlighting their potential as advanced wound dressing materials with controlled antimicrobial delivery.

## 1. Introduction

It is well known that hydrogels are suitable for biomedical applications such as tissue engineering, wound healing, and controlled drug delivery due to their porosity, biocompatibility, and mechanical and physicochemical properties [1,2,3,4,5,6,7]. The design of multifunctional hydrogels with mechanical stability, biocompatibility, and antibacterial properties remains a major focus of current biomaterials research [8]. Among the different polymer systems investigated, poly(vinyl alcohol) (PVA) and sodium alginate have emerged as complementary materials that can be combined to obtain hybrid hydrogels with optimized performance [9,10,11,12,13,14,15,16,17].

Pure PVA hydrogels often lack biological functionality and biodegradability, limiting their direct application in tissue engineering or wound care [12]. Consequently, PVA is frequently mixed with different polymers or bioactive compounds. Blending improves biocompatibility and introduces functional groups that enhance cell adhesion and proliferation [12,13]. For instance, hybrid hydrogels combining PVA with polysaccharides or proteins have been shown to improve water uptake and provide better integration with biological tissues [14,15]. The PVA matrix also serves as an excellent carrier for antibiotics, growth factors, and antibacterial metal nanoparticles due to its uniform microstructure and chemical stability [15,16].

Alginate hydrogels exhibit high biocompatibility, mild gelation conditions, and biodegradability, which make them suitable for the encapsulation of cells and drugs [4,18]. Nevertheless, pure alginate gels exhibit poor mechanical strength and limited stability under physiological conditions, particularly in the absence of crosslinking ions [19,20]. To address these shortcomings, alginate is often combined with synthetic polymers like PVA to form interpenetrating polymer networks or semi-interpenetrating hydrogels [21]. Such hybrid structures exhibit improved elasticity, thermal stability, and controlled swelling behavior while maintaining the favorable biological features of alginate [1,22,23,24].

Silver is well known for its broad-spectrum antibacterial, antifungal, and antiviral properties, acting through multiple mechanisms [25,26]. Incorporating silver nanoparticles (AgNPs) into composite PVA–alginate hydrogels could enable the development of improved antimicrobial biomaterials for potential applications in wound dressings and tissue engineering [24,27,28,29,30,31]. In situ synthesis of AgNPs within the hydrogel matrix typically results in uniform nanoparticle distribution and strong interaction with the polymer chains, enhancing both mechanical stability and antimicrobial activity [2,26,29,32,33,34]. In contrast, the ex situ addition of pre-synthesized nanoparticles allows for better control over particle size but may lead to agglomeration or uneven dispersion [35].

The synergistic combination of PVA and alginate provides a versatile platform for fabricating hydrogels with tailored mechanical, swelling, and biological properties. The incorporation of silver nanoparticles further expands the possibility to apply them in wound dressings, tissue regeneration, and antimicrobial coatings for implants [36,37,38]. Current research is focused on optimizing the crosslinking method, polymer ratio, and silver content to tune degradation rate, ion release profile, and cellular response [39,40,41,42].

In pharmacokinetics, a popular choice to describe the drug release is that of compartmental models like the general fractional derivative (GFD) model recently formulated by our group [43,44]. In this work, we studied the silver release behavior of electrochemically synthesized PVA–alginate hydrogels with embedded AgNPs using two compartmental GFD models and compared it with Korsmeyer–Peppas [45], Makoid–Banakar [46], Kopcha [47] and early-time approximation (ETA) [48,49] diffusion models.

The novelty of the present study is the novel general fractional derivative (GFD) model to investigate silver release from AgNP-loaded PVA–alginate hydrogels. To the best of our knowledge, this is the first application of the GFD model to alginate-based hydrogel systems. In contrast to conventional diffusion models, the GFD approach accurately describes the entire release profile, provides the lowest fitting residuals, and enables the determination of the effective silver diffusion coefficient over the complete release period throughout the entire release process, revealing the influence of alginate concentration on silver transport within the hydrogel network. These findings provide new insights into the structure–diffusion–function relationship of silver-loaded PVA–alginate hydrogels and offer a robust mathematical framework for the design and optimization of controlled silver-releasing wound dressings.

## 2. Results and Discussion

The electrochemical synthesis of silver nanoparticles within the hydrogel takes place by the reduction of Ag+ ions under constant applied voltage. Therefore, AgNPs are stabilized inside the hydrogel matrix through the interactions with polymer chains and by penetrating pores of the matrix, enabling long-term stabilization. In order to determine the optimal values of applied voltage (*U*) and time of synthesis (*t*), UV–visible spectroscopy was used. The parameters that were used to evaluate the optimal values *U* and *t* were the positions and intensities of absorption maxima (*λ*_max_ and *A*_max_, respectively), as well as nanoparticle diameters (*D*), estimated based on SPR peaks [50,51]. Varying the applied voltage between 50 and 110 V and time between 2 and 6 min, the lowest AgNP diameter was observed at 90 V and a time of 4 min; these parameters were chosen as optimal since it is well known that smaller AgNPs exhibit a stronger antibacterial effect. We have determined the same values of applied voltage and time earlier for different silver-loaded hydrogels [29,34,52,53,54,55]. Under these conditions, preferential accumulation of metallic silver may occur at the cathodic interface, which adversely affects nanoparticle formation efficiency. To counteract this phenomenon and promote nanoparticle generation, the direction of the applied electric field was periodically switched at 60 s intervals.

### 2.1. UV–Visible Spectroscopy

Since amounts of alginate were varied, two different colloid dispersions were obtained—P0.5A (PVA/0.5 wt% alginate) and P1.0A (PVA/1.0 wt% alginate). The incorporation of silver nanoparticles into the two hydrogel compositions resulted in samples designated SP0.5A (silver/PVA/0.5 wt% alginate) and SP1.0A (silver/PVA/1.0 wt% alginate). Silver nanoparticles were detected in SP1.0A and SP0.5A hydrogels by UV–Vis-spectroscopy, showing a strong optical peak near 400 nm due to surface plasmon resonance (SPR) [56]. Characteristic peak at 390–440 nm proves the presence of AgNPs inside the hydrogel matrix [57].

**Figure 1:**
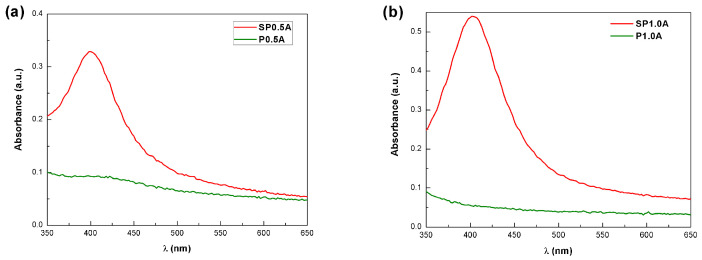
UV–Vis spectra of (**a**) P0.5A and SP0.5A hydrogels and (**b**) P1.0A and SP1.0A hydrogels.

The UV–Vis spectra of P1.0A, P0.5A, SP1.0A, and SP0.5A hydrogels are presented in Figure 1. All measurements for the hydrogel samples were expressed as averages obtained from three replicates. The absorption maxima observed at 400 nm for SP0.5A (Figure 1a) and 402 nm for SP1.0A (Figure 1b) proved the incorporation of spherical AgNPs with diameters of a few tens of nanometers. Namely, based on the published data for different silver-loaded hydrogels, it is well known that UV–Vis analysis has shown that the surface plasmon absorption band peaking in the wavelength range of 405–440 nm corresponds to silver nanoparticles with radii smaller than ∼30 nm [27,28,56,58]. These findings were confirmed by transmission electron microscopy (TEM) and dynamic light scattering (DLS) measurements for different hydrogels with embedded silver nanoparticles [29,30,34,53,55,59,60].

The UV–Vis spectra of the samples without silver, P0.5A (Figure 1a) and P1.0A (Figure 1b) did not exhibit peaks at ~400 nm. A higher absorbance of SP1.0A hydrogel (0.494 a.u.) than absorbance of SP0.5A (0.345 a.u.) proves that an increase in AgNP content was detected with rising alginate concentration in the hydrogel matrices due to a greater number of alginate functional groups capable of interacting with silver species.

### 2.2. FTIR Spectroscopy

**Figure 2:**
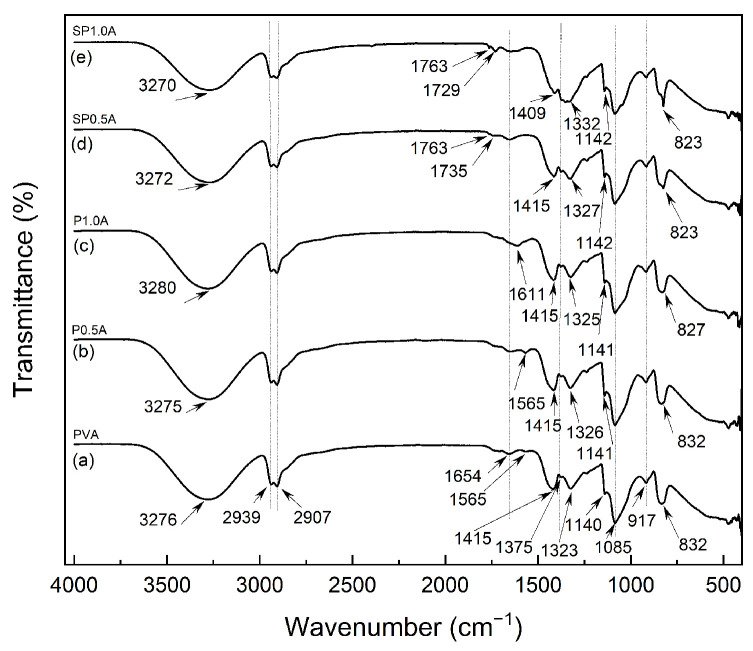
FTIR spectra of (**a**) PVA, (**b**) P0.5A, (**c**) P1.0A, (**d**) SP0.5A and (**e**) SP1.0A hydrogels.

Figure 2 illustrates the FTIR spectra of PVA, P0.5A, P1.0A, SP0.5A, and SP1.0A hydrogels.

Well-defined, broad absorption bands assigned to the stretching vibrations of hydrogen-bonded hydroxyl groups can be revealed in the interval from 3270 to 3280 cm^−1^ for all samples. A shift of bands’ positions to the lower wavenumbers can be observed after silver introduction to the hydrogels, e.g., from 3275 cm^−1^ (P0.5A) to 3272 cm^−1^ (SP0.5A) and from 3280 cm^−1^ (P1.0A) to 3270 cm^−1^ (SP1.0A), which further corroborates the interaction between silver nanoparticles and the hydroxyl functional groups present in the PVA and alginate polymer chains. Namely, upon the incorporation of silver nanoparticles, the hydroxyl stretching band underwent a slightly larger shift in the hydrogel containing 1.0 wt.% alginate than in the hydrogel containing 0.5 wt.% alginate, relative to their respective silver-free counterparts. Specifically, the band shifted by approximately 10 cm^−1^ for the P1.0A/SP1.0A pair, whereas a shift of only 3 cm^−1^ was observed for the P0.5A/SP0.5A pair. This greater band displacement at higher alginate content suggests that the higher availability of hydroxyl functional groups promotes the formation of a larger number of interactions between the silver nanoparticles and the PVA/alginate network.

For all hydrogels, bands at 2939 cm^−1^ and 2907 cm^−1^ are assigned to C-H stretching vibration in both alginate and PVA structures [61,62]. In pure alginate, a band detected at 1712 cm^−1^ is assigned to the H–O–C=O stretching vibrations [27]. For Ag-containing hydrogels, bands at 1763 and 1735 cm^−1^ for SP0.5A and at 1763 and 1729 cm^−1^ for SP1.0A were observed (Figure 2). Perhaps it can be assumed that significant bands’ shifting to the higher wavenumbers can be consequences of additional bonds established between silver particles and the H–O–C=O group, confirming the interaction between polymers and Ag particles. At 1654 cm^−1^, the presence of PVA-absorbed water could be detected in all samples. At 1565 cm^−1^, the –OH bending vibration in pure PVA hydrogel, as well as in the hydrogel with a smaller amount of alginate (P0.5A), can be noticed [62]. According to the literature [63], it was reported that the band detected at 1610 cm^−1^ can be assigned to the CO stretching in the carbonyl group, belonging to the alginate structure. Previously mentioned functional groups can create hydrogen bonding between PVA and alginate. Due to more hydrogen bonds forming between polymers, e.g., intra- and inter-molecular interaction, the band at 1611 cm^−1^ can be detected in the case of a higher alginate amount in hydrogel (P1.0A) (Figure 2). For pure PVA, P0.5A, P1.0A and SP0.5A, bands at 1415 cm^−1^ can be observed, while the higher concentration of alginate in combination with PVA and silver, SP1.0A, causes a slight shift of this band to the lower wavenumber, at 1409 cm^−1^. These bands represent the combination of FTIR bands for both PVA and alginate functional groups’ vibrations, assigned to the –OH in-plane coupling with C–H wagging in the CH_2_ group in the PVA structure and the symmetric stretching vibration of the COO- groups in the alginate structure [61,64]. Based on the bands’ shift, it can be proposed that an increase in alginate concentration in Ag-containing hydrogels increases the alginate impact on the band position, as seen by a slight shift to the lower wavenumber (1409 cm^−1^). This indicates the interactions of embedded silver inside the polymer matrix, in particular with the O–H group of both PVA and alginate. For all hydrogels, the FTIR bands detected at around 1375 cm^−1^ can be assigned to the CH_2_ wagging in the PVA structure (Figure 2) [62]. The band at 1323 cm^−1^ aligns well with the C–H wagging of pure PVA hydrogel, while for all multicomponent hydrogels, the presence of this band can be detected in the range from 1325 to 1332 cm^−1^, suggesting the interactions between the components. A weak band, originating from symmetric stretching of C–O, could be detected at 1140 cm^−1^ for pure PVA hydrogel. The existence of this band, in all analyzed hydrogels, proves the crystalline sequence of PVA [55,62]. FTIR bands, detected at 1085 cm^−1^ in all hydrogels, can be assigned to the C–O stretching in secondary alcohols (PVA, C–O–H group), overlapping the C–C and C–O stretching in alginate structure [61,62]. A bend at around 917 cm^−1^, observed for all hydrogels, can be assigned to the rocking of the CH_2_ vibration in the PVA structure [62]. For pure PVA hydrogel, a band corresponding to the C–C stretching in the PVA structure can be detected at 832 cm^−1^ (Figure 2) [62]. Corresponding FTIR bands for P0.5A and P1.0A hydrogels are revealed at 832 and 827 cm^−1^, respectively, suggesting that a smaller amount of alginate (0.5% in P0.5A) does not influence the FTIR band position of PVA, while a higher amount of alginate (1.0% in P1.0A) causes a slight shift to the lower wavenumber, suggesting a more intensive interaction between the two polymers (PVA and Alg). Silver introduction to PVA/Alg hydrogels leads to the appearance of significantly sharper FTIR bands at lower wavenumbers, e.g., at 823 cm^−1^ for both SP0.5A and SP1.0A hydrogels, indicating the silver interaction with polymers, improving the stability of silver particles in the silver-doped polymer hydrogels.

FTIR spectroscopy revealed the formation of interactions between alginate chains and silver nanoparticles, confirming the suitability of alginate for use in the electrochemical synthesis of AgNPs. Alginate additionally contributes to nanoparticle stabilization due to the presence of hydroxyl (–OH) and carboxylate (–COO^−^) functional groups. The interactions between alginate and silver nanoparticles, as evidenced by shifting of the characteristic vibrational bands associated with hydroxyl (–OH) and carboxylate (–COO^−^) groups, indicate their involvement in nanoparticle stabilization. Alginate is widely recognized as an effective stabilizing agent for AgNPs due to the interaction of characteristic groups with the Ag nanoparticle surface, reducing particle aggregation [27]. The influence of another polymer component, poly(vinyl alcohol), on the incorporation and stabilization of AgNPs within the PVA/alginate hydrogel matrix can be explained by the formation of complex bonds between Ag+ ions and electron pairs on the oxygen atoms of the hydroxyl groups in PVA, facilitating the silver ion reduction and improving nanoparticle dispersion within polymer matrices [26]. Consequently, the combined presence of alginate and PVA contributes to the stabilization and homogeneous distribution of AgNPs within the Ag/PVA/Alg hydrogel network.

### 2.3. Evaluation of Antibacterial Properties

The antibacterial efficiency of P1.0A, P0.5A, SP1.0A and SP0.5A hydrogels was evaluated by monitoring bacterial growth kinetics in the presence of hydrogel samples against *Staphylococcus aureus* and *Escherichia coli*, which are among the most prevalent pathogens associated with wound infections.

**Figure 3:**
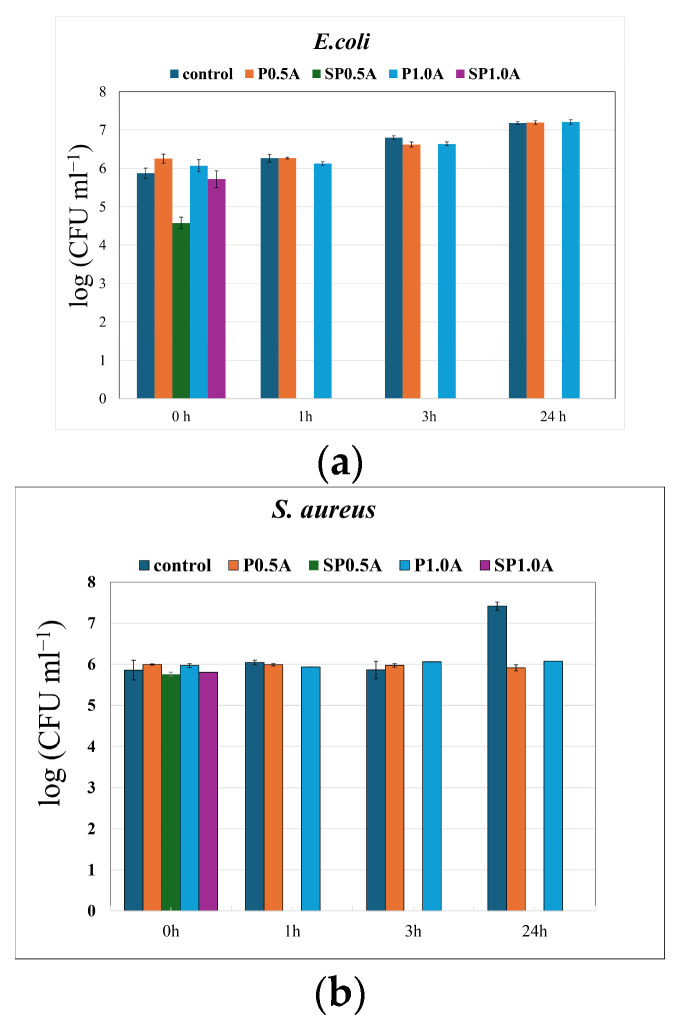
Results of the antibacterial activity tests of P0.5A, SP0.5A, P1.0A, and SP1.0A hydrogels against the bacterial strains (**a**) *Escherichia coli* ATCC25922 and (**b**) *Staphylococcus aureus* TL.

Figure 3 represents the quantification of surviving bacterial cells in the presence of P1.0A, P0.5A, SP1.0A and SP0.5A hydrogels. After 1 h, SP0.5A and SP1.0A hydrogels caused complete reduction of bacterial cells for both *S. aureus* and *E. coli*. The antibacterial activity of the investigated hydrogels against *Escherichia coli* was evaluated after 24 h of incubation. One-way ANOVA revealed a highly significant effect of the tested specimens on bacterial growth (Figure 3a). Tukey’s post hoc multiple comparison test demonstrated that the Ag-containing hydrogels (SP0.5A and SP1.0A) significantly inhibited bacterial growth compared with the control and the corresponding silver-free hydrogels (P0.5A and P1.0A) (*p* < 0.0001). In contrast, no significant differences were observed between the control and the silver-free hydrogels, nor between the two Ag-containing hydrogels (*p* > 0.05). Complete inhibition of *E. coli* growth was observed for both Ag-containing hydrogels after 24 h, as no viable colonies were detected.

One-way analysis of variance (ANOVA) demonstrated a statistically significant effect of the tested specimens on the growth of *S. aureus* after 24 h (Figure 3b). Tukey’s post hoc multiple comparison test demonstrated that all hydrogel formulations significantly reduced bacterial growth compared with the untreated control (*p* < 0.0001). However, no statistically significant differences were observed among the hydrogel formulations themselves (*p* > 0.05). Complete inhibition of bacterial growth was observed for the SP0.5A and SP1.0A hydrogels after 24 h, as no viable bacterial colonies were detected in any of the independent experiments.

The markedly stronger antimicrobial performance of AgNPs compared to bulk silver is attributed to their unique physicochemical behavior and biological reactivity. Although the mechanism has not been fully clarified, current understanding points to two closely linked pathways. One involves the direct interaction between nanoparticles and the bacterial surface, while the other is associated with the continuous release of biologically active silver ions [65].

AgNPs can breach the bacterial envelope, which leads to structural disruption, leakage of intracellular material, and ultimately cell death. Their nanoscale dimensions further amplify this effect, since smaller particles provide a substantially larger contact interface with microbial cells. After entering the cell, AgNPs can associate with vital components—including membranes, proteins, lipids, enzymes, ribosomes, and nucleic acids—triggering extensive cellular malfunction that ends in apoptosis [66].

In addition, AgNPs may stimulate excessive generation of reactive oxygen species (ROS) and free radicals while simultaneously impairing the bacterial antioxidant defense system. Their large surface area also enables a sustained release of Ag^+^ ions, which can interfere with ATP synthesis and disrupt essential cellular functions by binding to membrane-bound proteins or interacting with DNA [66,67]. Taken together, these combined effects—direct nanoparticle–cell interactions and ion-mediated toxicity—underlie the pronounced antibacterial potency of silver nanoparticles. Overall, the results demonstrate that the proposed wound dressing materials exhibit significant potential for use in antibacterial applications.

### 2.4. Cytotoxicity

**Figure 4:**
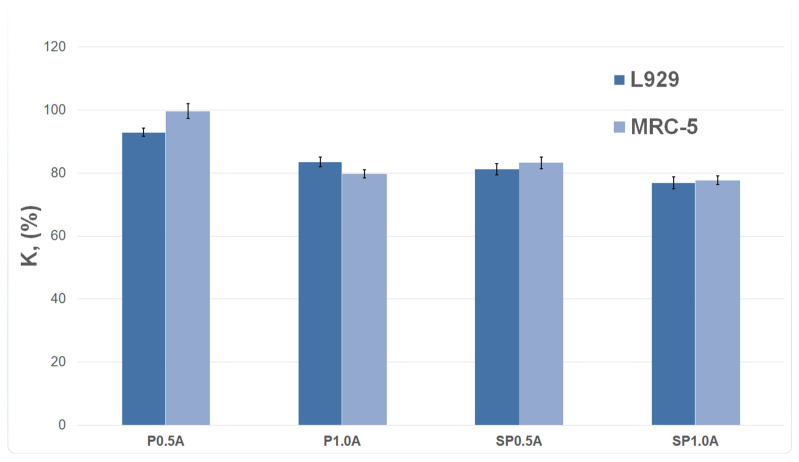
Cell viability of the L929 and MRC-5 cell lines in the presence of the hydrogel samples P0.5A, P1.0A, SP0.5A, and SP1.0A obtained by the DET assay.

**Figure 5:**
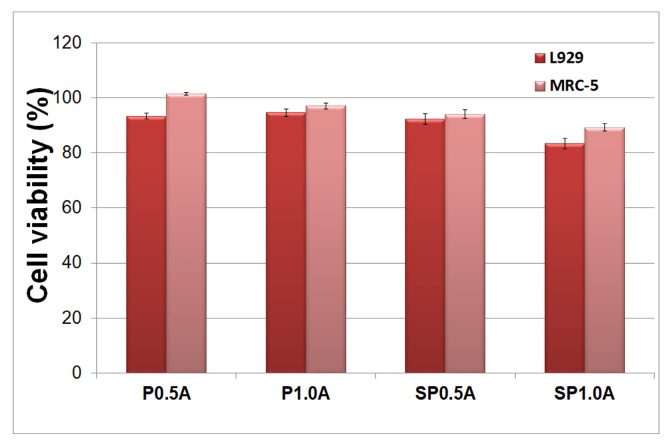
Cell viability of the L929 and MRC-5 cell lines in the presence of the hydrogel samples P0.5A, P1.0A, SP0.5A, and SP1.0A obtained by the MTT assay.

The cytotoxicity of the samples P0.5A, P1.0A, SP0.5A, and SP1.0A toward the L929 (mouse fibroblast) and MRC-5 (human fibroblast) cell lines was assessed using the DET and MTT assays. Figure 4 and Figure 5 present the results of the tests, showing cell viability relative to control. Data are presented as mean ± SD (*n* = 3). Statistical significance was determined by one-way ANOVA followed by Tukey’s post hoc test (*p* < 0.05). According to the cytotoxicity scale interpretation [68], the samples do not exhibit cytotoxic effects toward L929 and MRC-5.

The cytotoxicity of the hydrogel samples was assessed by dye-exclusion assay. This method relies on the fact that trypan blue selectively enters only damaged or non-viable cells, while living cells with intact membranes remain unstained [69]. Cell viability was calculated in respect to control and shown in Figure 4.

Both cell lines maintained high viability for all tested samples, indicating the absence of pronounced cytotoxic effects. As cell survival remained above 85% for all samples, the investigated hydrogels can be classified as non-cytotoxic.

The MTT assay results, shown in Figure 5, represent the percentage of viable cells compared with the control group (cells cultured without any sample). Based on the established cytotoxicity criteria, none of the tested materials demonstrated toxic effects. In fact, the MTT data indicated that the P0.5A, P1.0A, SP0.5A, and SP1.0A hydrogels exhibited non-cytotoxic effects against both MRC-5 and L929 cell lines. One-way ANOVA showed no statistically significant differences in cell viability among the tested hydrogel formulations for either the L929 cells or the MRC-5 cells. The conclusions regarding cytocompatibility were based primarily on the MTT results, supported by the observations from the DET assay since MTT is often used as the primary quantitative method for assessing cell metabolic activity, while the DET assay provides qualitative visualization of viable and non-viable cells. In the presented case this was strongly supported by statistical analysis (Figure 5). The MTT assay results indicate that mouse fibroblasts are more sensitive to the samples. Samples with a higher content of silver nanoparticles—specifically SP1.0A (as confirmed by UV-Vis spectroscopy)—lead to reduced cell viability.

### 2.5. Silver Release

**Figure 6:**
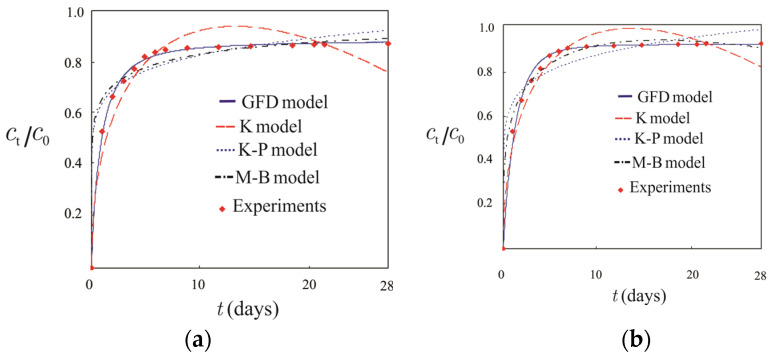
The *c*_t_/*c*_0_ values determined by GFD, M-B, K-P, and K models and experimental values for (**a**) SP0.5A and (**b**) SP1.0A hydrogels.

The experimental curves for SP0.5A and SP1.0A hydrogels (red points at Figure 6a and Figure 6b, respectively, presented as averages of three samples) were obtained by AAS. They indicated the silver initial burst release, favorable for efficient inhibition of bacterial growth, followed by slow release during a longer time period. Silver release experiments confirmed higher initial silver concentration in SP1.0A (417.37 mg dm^−3^) than in SP0.5A (382.60 mg dm^−3^), as was indicated also by UV-Vis spectra (Figure 1). To investigate the mechanism of silver release, experimental profiles were studied using different diffusion models as follows.

#### General Fractional Derivative Model

The classical two-compartmental system is described by a system of two equations [70]:

$$\frac{dm_{1}}{dt}=-k\left(\frac{m_{1}\left(t\right)}{V_{1}}-\frac{m_{2}\left(t\right)}{V_{2}}\right)+f_{1}\left(t\right),$$

(1) $$\frac{dm_{2}}{dt}=k\left(\frac{m_{1}\left(t\right)}{V_{1}}-\frac{m_{2}\left(t\right)}{V_{2}}\right)+f_{2}\left(t\right),$$

where *m*_1_ is the mass of silver in the hydrogel and *m*_2_ is the mass of released silver, *k* denotes the diffusion coefficient, *V*_1_ is the hydrogel volume, and *V*_2_ is the volume of the solution, while *f*_1_ and *f*_2_ are production terms. Note that in Equation (1), Fick’s model of diffusion across compartments’ boundaries is assumed.

In our earlier work [44,71], we proposed a generalization of the system (1) as:

bDtα,λ0Cm1t+aD¯tβ,Λ0Cm1t=−km1tV1−m2tV2+f1t,

(2) bDtα,λ0Cm2t+aD¯tβ,Λ0Cm2t=km1(t)V1−m2(t)V2+f2t,

where

Dtα,λ0Cm1t=bαΓ1−α∫0texp−λττα m11t−τdτ,   0 <α<1,

is the general fractional derivative of order α and

(3) D¯tβ,Λ0Cm1t=∫01aβΓ(1−β)∫0texp−Λττβm11t−τdτdβ,

is the distributed order of the general fractional derivative; a and b are non-negative constants, having dimensions of time related to relaxation time; α is a constant; λ>0 and *Λ* > 0 are constants; and *k* is a constant connected with the diffusion coefficient. Therefore, the expanded form of the system that we use is

$$\frac{b}{\Gamma \left(1-\alpha \right)}\int_{0}^{t}b^{\alpha -1}\;\frac{\exp\left(-\Lambda \tau \right)}{\tau^{\alpha }}{m_{1}}^{\left(1\right)}\left(t-\tau \right)d\tau +\;a\int_{0}^{1}a^{\beta -1}\left[\frac{1}{\Gamma \left(1-\beta \right)}\int_{0}^{t}\frac{\exp\left(-\lambda \tau \right)}{\tau^{\beta }}{m_{1}}^{\left(1\right)}\left(t-\tau \right)d\tau \right]d\beta \;=-k\left(\frac{m_{1}\left(t\right)}{V_{1}}-\frac{m_{2}\left(t\right)}{V_{2}}\right)+f_{1}\left(t\right),$$

(4) $$\frac{b}{\Gamma \left(1-\alpha \right)}\int_{0}^{t}b^{\alpha -1}\;\frac{\exp\left(-\Lambda \tau \right)}{\tau^{\alpha }}{m_{2}}^{\left(1\right)}\left(t-\tau \right)d\tau +\;a\int_{0}^{1}a^{\beta -1}\left[\frac{1}{\Gamma \left(1-\beta \right)}\int_{0}^{t}\frac{\exp\left(-\lambda \tau \right)}{\tau^{\beta }}{m_{2}}^{\left(1\right)}\left(t-\tau \right)d\tau \right]d\beta \;=k\left(\frac{m\left(t\right)}{V_{1}}-\frac{m_{2}\left(t\right)}{V_{2}}\right)+f_{2}\left(t\right).$$

The initial conditions corresponding to Equation (4) are

(5) $$m_{1}(0)=m_{0},\;\;\;\;m_{2}(0)=0.$$

In [41,42], we solved system Equations (4) and (5) by using Laplace transform. The result is

$${\hat{m}}_{2}(s)=\frac{1}{V_{1}}\frac{k\left(m_{0}+\frac{{\hat{f}}_{1}\left(s\right)+{\hat{f}}_{2}\left(s\right)}{\frac{b}{{\left[b\left(s+\lambda \right)\right]}^{1-\alpha }}+bK\left(s\right)}\right)}{s\left[\frac{bs}{{\left[b\left(s+\Lambda \right)\right]}^{1-\alpha }}+asK\left(s\right)+k\left(\frac{1}{V_{1}}+\frac{1}{V_{2}}\right)\right]},$$

(6) $${\hat{m}}_{1}\left(s\right)=\frac{m_{0}}{s}-\frac{{\hat{f}}_{1}\left(s\right)+{\hat{f}}_{2}\left(s\right)}{s\left[\frac{b}{{\left[b\left(s+\Lambda \right)\right]}^{1-\alpha }}+bK\left(s\right)\right]},$$

where ${\hat{m}}_{2}\left(s\right)$ denotes the Laplace transform and

$$K\left(s\right)=\int_{0}^{1}\frac{d\beta }{{\left[a\left(s+\lambda \right)\right]}^{1-\beta }}.$$

From Equation (6), by using the inverse Laplace transform and introducing relative masses

$$q_{1}(t)=\frac{m_{1}\left(t\right)}{m_{0}},\;\;\;\;\;\;\;q_{2}(t)=\frac{m_{2}(t)}{m_{0}},$$

it follows

$$q_{1}(1)=1-q_{2}(t),$$

(7) $$q_{2}(t)=\underset{P\to \infty }{\lim}\frac{k}{V_{1}}\frac{1}{2\pi }\int_{x_{0}-iP}^{x_{0}+iP}\frac{\exp(x_{0}+ip)t}{\left(x_{0}+ip\right)\left[\frac{b\left(x_{0}+ip\right)}{{\left[b\left(x_{0}+ip+\lambda \right)\right]}^{1-\alpha }}+a\left(x_{0}+ip\right)K\left(\left(x_{0}+ip\right),\lambda \right)+k\left(\frac{1}{V_{1}}+\frac{1}{V_{2}}\right)\right]}dp.$$

Note that $q_{1}\left(t\right)+q_{2}\left(t\right)=1$. The equations in Equation (7) are solutions of the general fractional derivative model (GFD) presented in (7). The values of the parameters a,k,λ,b,β,Λ are calculated by using the least square method.

The silver release experimental profiles determined by atomic absorption spectroscopy are presented (Table 1) as the ratio between the concentration of silver released at time *t*, *c*_t_, and the initial silver concentration in the hydrogel, *c*_o_. We note that $c_{t}=m_{2}/V_{1}$ and $c_{0}=m_{0}/V_{1}$, so $c_{t}/c_{0}=m_{2}/m_{0}=q_{2}$.

Let the square residual, *R*, be defined as

$$R(a,k,\lambda ,b,\beta ,\lambda )=\sum_{j=1}^{14}{\left[\frac{C_{t}}{C_{0}}(t_{j})-{\left(\frac{C_{t}}{C_{0}}\right)}_{m}(t_{j})\right]}^{2},$$

where $c_{t}/c_{0}(t_{j})=m_{2}/m_{0}=q_{2}(t_{j})$ denotes the values calculated using Equation (7), while ${\left(c_{t}/c_{0}\right)}_{m}(t_{j})$ represents experimental values at time *t*_j_. Parameters a and b must be

(8) a>0, b>0.

The constant *k* is connected with the diffusion coefficient, so k>0, and

(9) λ≥0,  Λ≥0.

In order to obtain k in the dimension cm^2^/s, we multiplied both sides of Equation (4) with 1/day. From the condition

(10) $$\underset{a,k,\lambda ,b,\beta ,\Lambda }{\min}R(a,k,\lambda ,b,\beta ,\Lambda ),$$

The parameter values for SP0.5A hydrogel were calculated to be

$$a=0.00008797\;{day}^{1},\;k=0.000852\;\frac{{cm}^{5\;}}{day},\;\;\;\lambda =0.00504\;{day}^{-1},$$

$$b=2.8495930774\;{day}^{1},\;\;\beta =0.823,\;\;\Lambda =0.2933\;{day}^{-1},\underset{a,k,\lambda ,b,\beta ,\Lambda }{\min}R(a,k,\lambda ,b,\beta ,\Lambda )=0.0013896.$$

Similarly, the parameters values for SP1.0A hydrogel were calculated to be

$$a=0.0000022852\;{day}^{1},\;k=0.0006183807\;\frac{{cm}^{5}}{day},\;\lambda =0.0000001133\;{day}^{-1}$$

$$b=2.0841381888\;{day}^{1},\;\;\;\beta =0.9507,\;\;\Lambda =0.1134327942\;{day}^{-1},\;\underset{a,k,\lambda ,b,\beta ,\Lambda }{\min}R(a,k,\lambda ,b,\beta ,\Lambda )=0.00513664.$$

The parameter values calculated from the GFD model and quality of fitting, R, for SP0.5A and SP1.0A hydrogels are presented in Table 2.

To obtain the diffusion coefficient, *D*, from Equation (2), we introduce a characteristic length ∆ [71] in Equation (4) so that

$$k\left(\frac{m_{1}\left(t\right)}{V_{1}}-\frac{m_{2}\left(t\right)}{V_{2}}\right),$$

is replaced with

$$\frac{k\left(\frac{m_{1}\left(t\right)}{V_{1}}-\frac{Q_{m}\left(t\right)}{V_{2}}\right)}{\Delta },$$

where ∆ has the dimension of length. Therefore, the expression for the diffusion coefficient becomes $D=\frac{k/∆}{A},$ where A is the area over which diffusion takes place. Since we are using the samples in the form of a cylinder (radius, r=11.5 mm; thickness, δ=4.5 mm), the surface area is $A=2r^{2}\pi +2r\pi \delta =11.561\;{cm}^{2}$. With ∆=0.015cm, we obtained $D=5.686\times 10^{-8}\;{cm}^{2}/s$ and $4.127\times 10^{-8}\;{cm}^{2}/s$ for SP0.5A and SP1.0A hydrogels, respectively (Table 2). A smaller value of the silver diffusion coefficient, i.e., a slower silver release for the SP1.0A hydrogel, can be interpreted by increased established bonds between silver nanoparticles and -O and -OH groups inside the PVA/Alg polymer matrix with higher alginate concentration and, consequently, more stable Ag-Alg complexes. These findings are in accordance with UV-Vis measurements, proving a greater amount of silver nanoparticles inside the matrix with higher alginate concentrations.

To elucidate the mechanisms governing silver release, the GFD model results were systematically compared with several theoretical diffusion models, enabling quantitative determination of relevant diffusion parameters. Models of Makoid–Banakar (M-B) [46], Korsmeyer–Peppas (K-P) [45], and Kopcha (K) [47] are defined by Equations (11), (12) and (13), respectively:

(11) $$\frac{C_{t}}{C_{0}}=k_{MB}\cdot t^{n}\cdot exp(-c\cdot t)$$

(12) $$\frac{C_{t}}{C_{0}}=k_{KP}\cdot t^{n}$$

(13) $$\frac{C_{t}}{C_{0}}=A\cdot t^{1/2}+B\cdot t$$

where *k*_MB_ and c are constants in the M-B model; *k*_KP_ is the K-P rate constant; n is the release exponent, and A and B are the K constants.

The resulting curves are shown in Figure 6a,b, for SP0.5A and SP1.0A hydrogels, respectively, indicating an initial burst release, with approximately 66% of the loaded silver nanoparticles released within the first 48 h, followed by a sustained, slower release over time. The model parameters and R for M-B, K-P and K models are shown in Table 2.

It is seen that the GFD model describes the silver release diffusion better than the other models since the calculated values of square residual from the GFD model are one order of magnitude lower than the values calculated from the other models (Table 2). Since n is less than 0.5, silver release is governed by Fickian diffusion [45]. This is in accordance with the K model, since ∣A∣ > ∣B∣, confirming that diffusion rather than polymer relaxation dominated the release process. The early time approximation (ETA) model [48] was used to calculate *D* from Equation (14) and compare it with *D* determined from the GFD model.

$$\frac{c_{t}}{c_{0}}=4\cdot {\left(\frac{D\cdot t}{\pi \cdot r^{2}}\right)}^{1/2}-\pi \cdot \left(\frac{D\cdot t}{\pi \cdot r^{2}}\right)-\frac{\pi }{3}\cdot {\left(\frac{D\cdot t}{\pi \cdot r^{2}}\right)}^{3/2}+4\cdot {\left(\frac{D\cdot t}{\pi \cdot \delta^{2}}\right)}^{1/2}$$

(14) $$-\frac{2r}{\delta }\cdot \left[8\cdot \left(\frac{D\cdot t}{\pi \cdot r^{2}}\right)-2\pi \cdot {\left(\frac{D\cdot t}{\pi \cdot r^{2}}\right)}^{3/2}-\frac{2\pi }{3}\cdot {\left(\frac{D\cdot t}{\pi \cdot r^{2}}\right)}^{2}\right]$$

**Figure 7:**
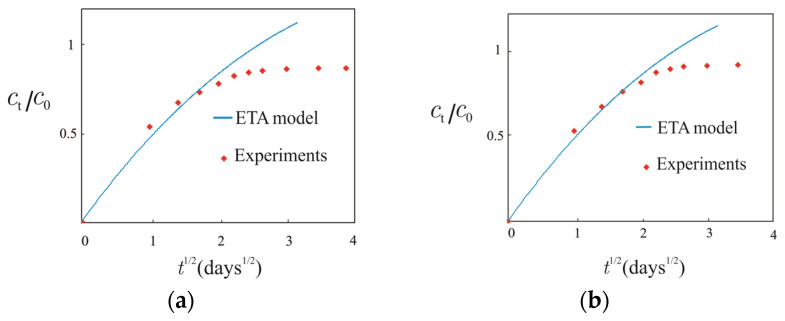
The *c*_t_/*c*_0_ values determined from the ETA model and experimental values for (**a**) SP0.5A and (**b**) SP1.0A hydrogels.

Figure 7 depicts the *c*_t_/*c*_0_ ratio as a function of the square root of time. We determined *D* in Equation (14) by minimizing square residual

$$Z(D)=\sum_{j=1}^{3}{\left[c_{t}/c_{0}(t_{j})-{\left(c_{t}/c_{0}\right)}_{m}(t_{j})\right]}^{2}$$

of three initial points to be $7.0066\times 10^{-8}\;{cm}^{2}/s$ and $5.686\times 10^{-8}\;{cm}^{2}/s$ for SP0.5A and SP1.0A hydrogels, respectively. A smaller *D* for the SP1.0A hydrogel was also obtained from the ETA model due to the increased number of established bonds between greater amounts of embedded AgNPs in the polymer matrix with greater alginate concentration, as was explained earlier for the GFD model. The differences between values of diffusion coefficients calculated from GFD and ETA models are derived from the time period taken in calculations. Since the silver diffusion coefficient from the GFD model was calculated for the entire time period, while *D* from ETA was only for the initial time period, we consider the *D* values obtained from the GFD model much more precise and relevant for diffusion studies.

## 3. Materials and Methods

### 3.1. Materials

Chemicals used in the experiments were: poly(vinyl alcohol) powder (fully hydrolyzed, Mw = 70–100 kDa, Sigma Aldrich (St. Louis, MO, USA), alginate powder (sodium alginate MW 10,000–600,000 g/mol, viscosity 350–550 mPas, AppliChem GmbH (PanReac AppliChem) (Darmstadt, Germany), silver nitrate (M = 169.87 gmol^−1^, Sigma Aldrich, USA), and potassium nitrate (M = 101.11 gmol^−1^, Alkaloid (Skopje, North Macedonia). All experiments were conducted using deionized water, purified by filtering distilled water using the GenPure ultrapure water system TKA GmbH (Niederelbert, Germany). Monobasic and dibasic potassium phosphates Centrohem (Stara Pazova, Serbia); Sigma-Aldrich (St. Louis, MO, USA)—needed for antibacterial assays. Cytotoxicity testing engaged a set of specific chemicals: MTT tetrazolium salt, fetal calf serum, EDTA, and antibiotic–antimycotic solution Sigma-Aldrich (St. Louis, MO, USA).

### 3.2. Electrochemical Synthesis of Ag/PVA/Alg Hydrogels

PVA powder was dissolved in hot distilled water at 90 °C for 2 h under magnetic stirring to yield colloid dispersion (10 wt%). Alginate colloid dispersions (0.5 wt% and 1 wt%) were also prepared by dissolving powder in dH_2_O at 90 °C for 2 h. Two solutions were then mixed gradually under continuous stirring to obtain uniform PVA–alginate dispersions. The amounts of alginate were varied, yielding two different colloid dispersions with 0.5 wt% alginate (sample marked P0.5A) and 1.0 wt% alginate (sample marked P1.0A). The gelation was then carried out by casting the colloid dispersions in Petri dishes and exposing them to five freezing (−18 °C for 16 h) and thawing (+4 °C for 8 h) cycles. Prior to electrochemical synthesis of silver nanoparticles (AgNPs), the P0.5A and P1.0A hydrogel discs (diameter 10 mm, height 5 mm) were swollen for 48 h in 0.25 mM AgNO_3_ (aq) solution in the dark. To enhance the ionic conductivity of the electrolyte, 0.1 M KNO_3_ was introduced.

To obtain samples embedded with Ag, silver nanoparticles were incorporated into hydrogel disc matrices through an in situ electrochemical approach at a constant voltage of 90 V using a laboratory-fabricated glass cell [48], yielding samples marked as SP0.5A and SP1.0A accordingly. The synthesis was conducted in a two-electrode arrangement with platinum electrodes (working and counter electrodes), connected to a DC power source (MA 8903 Electro-Phoresis, Iskra, Ljubljana, Slovenia). The total duration of the electrochemical treatment was 4 min, during which the polarity of the electrodes was changed at 60 s intervals to prevent excessive silver accumulation on the cathode surface. Prior to and following each experiment, the platinum electrodes were sequentially cleaned with a 1:1 (*v*/*v*) HNO_3_ solution and rinsed with distilled water.

### 3.3. UV-Vis and FTIR Spectroscopy

Ultraviolet–visible (UV–Vis) spectra were measured over the 350–650 nm wavelength range using a UV-3100 spectrophotometer (MAPADA, Shanghai, China). Fourier-transform infrared (FTIR) spectra were obtained using a Nicolet iS10 FTIR spectrometer (Thermo Fisher Scientific, Waltham, MA, USA) at a wavenumber range of 4000 cm^−1^ to 400 cm^−1^.

### 3.4. Atomic Absorption Spectroscopy

#### 3.4.1. Silver Release Study

SP0.5A and SP1.0A hydrogels were incubated in a chloride-free phosphate buffer (PB, pH 7.42) containing only KH_2_PO_4_ and K_2_HPO_4_ to avoid AgCl precipitation and maintained at 37 °C. The buffer solution containing released silver was collected periodically for 28 days, with buffer replacement every 24 h during the first week and every 3–5 days thereafter.

#### 3.4.2. Quantification of Released Silver

The concentrations of released silver were measured using a PYU UNICAM atomic absorption spectrophotometer Koninklijke Philips N.V., (Amsterdam, The Netherlands). Prior to analysis, a few drops of 1:1 (*v*/*v*) HNO_3_ were added to collected samples in order to convert any released silver nanoparticles into Ag^+^ ions. After 28 days, the samples were dissolved in 1:1 (*v*/*v*) HNO_3_ to determine residual silver remaining in the hydrogels.

All measurements were performed in triplicate to ensure reproducibility. Data were used to construct cumulative release profiles and to model silver diffusion kinetics using the selected mathematical models.

### 3.5. Evaluation of Antibacterial Activity

#### Kinetics of Antibacterial Activity

The hydrogels were first sectioned into fragments of approximately 2 × 2 mm. The obtained pieces were subsequently exposed to UV irradiation for 30 min inside a laminar airflow cabinet to achieve sterilization. Bacterial inocula were prepared from overnight cultures by resuspending the cells in phosphate buffer, identical to that employed in the swelling and drug-release studies, to obtain a starting density of approximately 1 × 10^6^ CFU/mL. Approximately 2 g of each sterilized hydrogel was then introduced into vessels containing the prepared bacterial suspensions, followed by incubation in a thermostatically controlled water bath at 37 °C. The bath was equipped with a shaker set to 62 rpm to ensure uniform temperature conditions. Control samples consisted of bacterial suspensions in phosphate buffer without added hydrogels. At predetermined time intervals (15 min, 1 h, 3 h, and 24 h), samples of the incubation medium were withdrawn and subjected to stepwise serial dilution for quantitative analysis. Aliquots (100 µL) of each diluted suspension were transferred into sterile Petri plates, followed by the addition of molten nutrient agar (1.5 wt%, maintained at approximately 55 °C). The plates were incubated at 37 °C for 24 h, after which the resulting colonies were counted. The number of viable microorganisms was calculated and reported as colony-forming units per milliliter (CFU/mL).

### 3.6. Cytotoxicity Assays

The cytotoxicity of P1.0A, P0.5A, SP1.0A, and SP0.5A hydrogels was evaluated using both the dye-exclusion test (DET) with trypan blue and the MTT assay. Two fibroblast cell lines were used: L929 (mouse-derived) and MRC-5 (human-derived). Approximately 10^5^ cells/mL were seeded into 12-well plates with hydrogel discs, while wells containing only cells served as controls. Plates were incubated at 37 °C in a 5% CO_2_ atmosphere for 48 h.

For the DET, cells were detached by trypsinization, and viable cells were counted using trypan blue exclusion. Cell viability was expressed as a percentage relative to control wells.

For the MTT assay, 100 μL of cell suspension per well was incubated for 48 h at 37 °C. MTT solution was added and incubated for 3 h at 37 °C. After removing the medium and MTT solution, the resulting formazan crystals were dissolved in 100 μL of 0.04 M HCl in isopropanol. Absorbance was measured at 540/690 nm using a Multiscan MCC340 spectrophotometric plate reader. Wells containing only MTT in culture medium were used as blanks. Growth inhibition was calculated relative to untreated control cells. All experiments were performed in triplicate.

### 3.7. Statistical Analysis

Statistical analysis was performed using one-way analysis of variance (ANOVA) to evaluate differences among experimental groups. When significant differences were detected, Tukey’s post hoc multiple comparison test was applied. Data are presented as mean ± standard deviation (SD) of at least three independent experiments, and differences were considered statistically significant at *p* < 0.05.

## 4. Conclusions

In conclusion, electrochemically synthesized silver embedded PVA–alginate hydrogels with different alginate concentrations (0.5 and 1.0 wt%) were shown to be non-cytotoxic toward MRC-5 and L929 cells and to exhibit strong antibacterial activity against *E. coli* and *S. aureus*. Analysis of silver release using a novel general fractional derivative (GFD) model versus previously well-established models revealed that GFD, since it has more parameters, better describes the silver release kinetics in the sense that the residual Equation (10) is smaller. In addition, it provided the possibility to determine the silver diffusion coefficient considering the complete release period. The presented results have shown the potential of studied hydrogels for applications in successful antibacterial wound dressings with controlled silver delivery.

## Figures and Tables

**Table 1:** The *c*_t_/*c*_0_ values for SP0.5A and SP1.0A hydrogels in phosphate buffer (PB) at pH 7.42 and 37 °C.

Time (Days)	*c*_t_/*c*_0_ (SP0.5A)	*c*_t_/*c*_0_ (SP1.0A)
0	0	0
1	0.530	0.523
2	0.666	0.662
3	0.727	0.751
4	0.775	0.805
5	0.820	0.860
6	0.839	0.883
7	0.849	0.894
9	0.855	0.901
12	0.859	0.905
15	0.862	0.909
19	0.865	0.911
21	0.867	0.913
22	0.869	0.915
28	0.871	0.917

**Table 2:** The model parameters and quality of fitting, R, for SP0.5A and SP1.0A hydrogels.

**SP0.5A**								
Parameters	*β*	*λ*_1_ (day^−1^)	*λ*_2_ (day^−1^)	*k* (cm^5^/day)	$D=\frac{k/∆}{A},$ (cm^2^/s)	*a* (day)	*b* (day)	R
GFD	0.823	0.00504	0.2933	0.0008522	5.686 × 10^−8^	0.00008797	2.733	0.0013896
Parameters	*k*_KP_ (day^−n^)			*n*		R		
K–P	0.641			0.1092		0.0301969		
Parameters	*k*_MB_ (day^−n^)			*n*		*C*	R	
M–B	0.6603			0.10791		0.002122	0.0272206	
Parameters	*A* (day^−1/2^)			*B* (day^−1^)		R		
K	0.5096			−0.0691		0.0480492		
**SP1.0A**								
Parameters	*β*	*λ*_1_ (day^−1^)	*λ*_2_ (day^−1^)	*k* (cm^5^/day)	$D=\frac{k/∆}{A}$ (cm^2^/s)	*a* (day)	*b* (day)	R
GFD	0.9507	1.133 × 10^−7^	0.1134	0.000618	4.127 × 10^−8^	0.00000228	2.0841	0.00513664
Parameters	*k*_KP_ (day^−n^)			*n*		R		
K–P	0.65034			0.12288		0.0436298		
Parameters	*k*_MB_ (day^−n^)			*n*		*C*	R	
M–B	0.60104			0.2394		0.01415	0.0106785	
Parameters	*A* (day^−1/2^)			*B* (day^−1^)		R		
K	0.52747			−0.07074		0.039048		

## Data Availability

The original contributions presented in this study are included in the article. Further inquiries can be directed to the corresponding author.

## References

[1] Thang N.H., Chien T.B., Cuong D.X. (2023). Polymer-Based Hydrogels Applied in Drug Delivery: An Overview. Gels.

[2] Nešović K., Mišković-Stanković V. (2020). A comprehensive review of the polymer-based hydrogels with electrochemically synthesized silver nanoparticles for wound dressing applications. Polym. Eng. Sci..

[3] Zhu Y., Cong H., Yu B. (2025). Current status and future developments in preparation and application of self-healing hydrogels for biomedical engineering. Eur. Polym. J..

[4] Liu M., Wang T., Wang X., Ren W., Zhang Y., Li L., Diao H. (2025). Multifunctional hydrogels: Therapeutic strategies and advances in inflammation. Eur. Polym. J..

[5] Gyles D.A., Castro L.D., Silva J.O.C., Ribeiro-Costa R.M. (2017). A review of the designs and prominent biomedical advances of natural and synthetic hydrogel formulations. Eur. Polym. J..

[6] Koehler J., Brandl F.P., Goepferich A.M. (2018). Hydrogel wound dressings for bioactive treatment of acute and chronic wounds. Eur. Polym. J..

[7] Farhadi M., Moghari S., Naqvi M., Khonakdar H.A. (2025). Recent Advances in Biodegradable Polymers for Wound Dressings, Infection, and Diabetes Treatment: A Review. Polym. Eng. Sci..

[8] Ferreira N.N., Ferreira L.M.B., Cardoso V.M.O., Boni F.I., Souza A.L.R., Gremião M.P.D. (2018). Recent advances in smart hydrogels for biomedical applications: From self-assembly to functional approaches. Eur. Polym. J..

[9] Li J., Mooney D.J. (2016). Designing hydrogels for controlled drug delivery. Nat. Rev. Mater..

[10] Wang T., Zhang F., Zhao R., Wang C., Hu K., Sun Y., Politis C., Shavandi A., Nie L. (2020). Polyvinyl Alcohol/Sodium Alginate Hydrogels Incorporated with Silver Nanoclusters via Green Tea Extract for Antibacterial Applications. Des. Monomers Polym..

[11] Zhang S., Han D., Ding Z., Wang X., Zhao D., Hu Y., Xiao X. (2019). Fabrication and Characterization of One Interpenetrating Network Hydrogel Based on Sodium Alginate and Polyvinyl Alcohol. J. Wuhan Univ. Technol. Mater. Sci. Ed..

[12] Liang X., Zhong H.-J. (2024). Polyvinyl Alcohol (PVA)-Based Hydrogels: Recent Progress in Fabrication, Properties, and Multifunctional Applications. Polymers.

[13] Jiang N., Qi B., Fan X., Yao L., Wang Y., Zhao Z., Xu Y., Razali M.H. (2023). Fabrication of biocompatible and biodegradable polyvinyl alcohol/sodium alginate blend polymers incorporating Ca^2+^ doped TiO_2_ nanocomposite 3D scaffold for biomedical applications. J. Saudi Chem. Soc..

[14] Xue H., Wang P., Ji L., Zhang K., Ge S., Tan J. (2025). Polysaccharide-based hydrogels: Materials, preparation, and applications in medicine, food, adsorption, and agriculture. J. Agric. Food Res..

[15] Rana M.M., De la Hoz Siegler H. (2024). Evolution of Hybrid Hydrogels: Next-Generation Biomaterials for Drug Delivery and Tissue Engineering. Gels.

[16] Filimon A., Dobos A.M., Onofrei M.D., Serbezeanu D. (2025). Polyvinyl Alcohol-Based Membranes: A Review of Research Progress on Design and Predictive Modeling of Properties for Targeted Application. Polymers.

[17] Sarvepalli S., Kandagatla H.P. (2025). Injectable hydrogel formulations: Design, synthesis and applications for subcutaneous delivery—A comprehensive review. Eur. Polym. J..

[18] Varaprasad K., Jayaramudu T., Kanikireddy V., Toro C., Sadiku E.R. (2020). Alginate-based composite materials for wound dressing application:A mini review. Carbohydr. Polym..

[19] Amponsah O., Nopuo P.S.A., Manga F.A., Catli N.B., Labus K. (2025). Future-Oriented Biomaterials Based on Natural Polymer Resources: Characteristics, Application Innovations, and Development Trends. Int. J. Mol. Sci..

[20] Wang Y., Shen Z., Wang H., Song Z., Yu D., Li G., Liu X., Liu W. (2025). Progress in Research on Metal Ion Crosslinking Alginate-Based Gels. Gels.

[21] Hamidi S., Monajjemzadeh F., Siahi-Shadbad M., Khatibi S.A., Farjami A. (2023). Antibacterial activity of natural polymer gels and potential applications without synthetic antibiotics. Polym. Eng. Sci..

[22] Chen X., Wu T., Bu Y., Yan H., Lin Q. (2024). Fabrication and Biomedical Application of Alginate Composite Hydrogels in Bone Tissue Engineering: A Review. Int. J. Mol. Sci..

[23] Kim Y.J., Min J. (2021). Property modulation of the alginate-based hydrogel via semi-interpenetrating polymer network (semi-IPN) with poly(vinyl alcohol). Int. J. Biol. Macromol..

[24] Iqbal Y., Amin F., Usman Y., Farrukh Sarfraz M. (2024). Alginate-Based hydrogels with inorganic Nanomaterials: A promising approach for wound healing and bone tissue regeneration. Eur. Polym. J..

[25] Paladini F., Pollini M. (2019). Antimicrobial silver nanoparticles for wound healing application: Progress and future trends. Materials.

[26] Nešović K., Mišković-Stanković V. (2022). Silver/poly(vinyl alcohol)/graphene hydrogels for wound dressing applications: Understanding the mechanism of silver, antibacterial agent release. J. Vinyl Addit. Technol..

[27] Jovanovic Ž., Stojkovska J., Bojana O., Miškovic-Stankovic V. (2012). Alginate hydrogel microbeads incorporated with Ag nanoparticles obtained by electrochemical method. Mater. Chem. Phys..

[28] Obradovic B., Stojkovska J., Jovanovic Z., Miskovic-Stankovic V. (2012). Novel alginate based nanocomposite hydrogels with incorporated silver nanoparticles. J. Mater. Sci. Mater. Med..

[29] Nešović K., Janković A., Radetić T., Vukašinović-Sekulić M., Kojić V., Živković L., Perić-Grujić A., Rhee K.Y., Mišković-Stanković V. (2019). Chitosan-based hydrogel wound dressings with electrochemically incorporated silver nanoparticles—In vitro study. Eur. Polym. J..

[30] Zvicer J., Miskovic-Stankovic V., Obradovic B. (2019). Functional bioreactor characterization to assess potentials of nanocomposites based on different alginate types and silver nanoparticles for use as cartilage tissue implants. J. Biomed. Mater. Res. Part A.

[31] Hu Q., Nie Y., Xiang J., Xie J., Si H., Li D., Zhang S., Li M., Huang S. (2023). Injectable sodium alginate hydrogel loaded with plant polyphenol-functionalized silver nanoparticles for bacteria-infected wound healing. Int. J. Biol. Macromol..

[32] Abdelmoneim H.M., Taha T.H., Alhudhaibi A.M., Afifi F.M., Faqihi A.A., Alsalamah S.A., Bendif H. (2025). Green Synthesis of Silver Nanoparticles and Polymeric Nanofiber Composites: Fabrications, Mechanisms, and Applications. Polymers.

[33] Ahmad N., Bukhari S.N.A., Hussain M.A., Ejaz H., Munir M.U., Amjad M.W. (2024). Nanoparticles incorporated hydrogels for delivery of antimicrobial agents: Developments and trends. RSC Adv..

[34] Nešović K., Janković A., Kojić V., Vukašinović-Sekulić M., Perić-Grujić A., Rhee K.Y., Mišković-Stanković V. (2018). Silver/poly(vinyl alcohol)/chitosan/graphene hydrogels—Synthesis, biological and physicochemical properties and silver release kinetics. Compos. Part B Eng..

[35] Duman H., Eker F., Akdaşçi E., Witkowska A.M., Bechelany M., Karav S. (2024). Silver Nanoparticles: A Comprehensive Review of Synthesis Methods and Chemical and Physical Properties. Nanomaterials.

[36] Zhu J., Cheng H., Zhang Z., Chen K., Zhang Q., Zhang C., Gao W., Zheng Y. (2024). Antibacterial Hydrogels for Wound Dressing. Gels.

[37] Jangid H., Singh S., Kashyap P., Singh A., Kumar G. (2024). Advancing biomedical applications: An in-depth analysis of silver nanoparticles in antimicrobial, anticancer, and wound healing roles. Front. Pharmacol..

[38] Dube E., Okuthe G.E. (2025). Silver Nanoparticle-Based Antimicrobial Coatings: Sustainable Strategies for Microbial Contamination Control. Microbiol. Res..

[39] Laib I., Gheraissa N., Benaissa A., Benkhira L., Azzi M., Benaissa Y., Abdelaziz A.G., Tian F., Walsh M., Bechelany M. (2025). Tailoring innovative silver nanoparticles for modern medicine: The importance of size and shape control and functional modifications. Mater. Today Bio.

[40] Harun-Ur-Rashid M., Foyez T., Krishna S.B.N., Poda S., Imran A. (2025). Bin Recent advances of silver nanoparticle-based polymer nanocomposites for biomedical applications. RSC Adv..

[41] Cao H., Duan L., Zhang Y., Cao J., Zhang K. (2021). Current hydrogel advances in physicochemical and biological response-driven biomedical application diversity. Signal Transduct. Target. Ther..

[42] Nanda D., Behera D., Pattnaik S.S., Behera A.K. (2025). Advances in natural polymer-based hydrogels: Synthesis, applications, and future directions in biomedical and environmental fields. Discov. Polym..

[43] Miskovic-Stankovic V., Janev M., Atanackovic T.M. (2023). Two compartmental fractional derivative model with general fractional derivative. J. Pharmacokinet. Pharmacodyn..

[44] Miskovic-Stankovic V., Atanackovic T.M. (2023). On a System of Equations with General Fractional Derivatives Arising in Diffusion Theory. Fractal Fract..

[45] Korsmeyer R.W., Gurny R., Doelker E., Buri P., Peppas N.A. (1983). Mechanisms of solute release from porous hydrophilic polymers. Int. J. Pharm..

[46] Makoid M.C., Dufour A., Banakar U.V. (1993). Modelling of dissolution behaviour of controlled release systems. STP Pharma Prat..

[47] Kopcha M., Lordi N.G., Tojo K.J. (1991). Evaluation of Release from Selected Thermosoftening Vehicles. J. Pharm. Pharmacol..

[48] Ritger P.L., Peppas N.A. (1987). A simple equation for description of solute release I. Fickian and non-fickian release from non-swellable devices in the form of slabs, spheres, cylinders or discs. J. Control. Release.

[49] Ritger P.L., Peppas N.A. (1987). A simple equation for description of solute release II. Fickian and anomalous release from swellable devices. J. Control. Release.

[50] Spasojevic J., Aleksandra Radosavljevic J.K., Mitric M., Popovic M., Rakocevic Z., Kalagasidis-Krusic M., Kacarevic-Popovic Z. (2017). Structural characteristics and bonding environment of Ag nanoparticles synthesized by gamma irradiation within thermo-responsive poly(N-isopropylacrylamide) hydrogel. Polym. Polym. Compos..

[51] Spasojević J., Milošević M., Vidičević-Novaković S., Tasić J., Milovanović P., Djurić M., Ranković D., Kačarević-Popović Z., Radosavljević A. (2024). Multifunctional Ag-Poly(N-isopropylacrylamide/itaconic Acid) Hydrogel Nanocomposites Prepared by Gamma Irradiation for Potential Application as Topical Treatment Dressings. Polymers.

[52] Abudabbus M.M., Jevremović I., Nešović K., Perić-Grujić A., Rhee K.Y., Mišković-Stanković V. (2018). In situ electrochemical synthesis of silver-doped poly(vinyl alcohol)/graphene composite hydrogels and their physico-chemical and thermal properties. Compos. Part B Eng..

[53] Jovanović Ž., Radosavljević A., Stojkovska J., Nikolić B., Obradovic B., Kačarević-Popović Z., Mišković-Stanković V. (2014). Silver/poly(N-vinyl-2-pyrrolidone) hydrogel nanocomposites obtained by electrochemical synthesis of silver nanoparticles inside the polymer hydrogel aimed for biomedical applications. Polym. Compos..

[54] Abudabbus M.M., Jevremović I., Janković A., Perić-Grujić A., Matić I., Vukašinović-Sekulić M., Hui D., Rhee K.Y.Y., Mišković-Stanković V. (2016). Biological activity of electrochemically synthesized silver doped polyvinyl alcohol/graphene composite hydrogel discs for biomedical applications. Compos. Part B Eng..

[55] Nešović K., Janković A., Perić-Grujić A., Vukašinović-Sekulić M., Radetić T., Živković L., Park S.J., Yop Rhee K., Mišković-Stanković V. (2019). Kinetic models of swelling and thermal stability of silver/poly(vinyl alcohol)/chitosan/graphene hydrogels. J. Ind. Eng. Chem..

[56] Slistan-Grijalva A., Herrera-Urbina R., Rivas-Silva J.F., Ávalos-Borja M., Castillón-Barraza F.F., Posada-Amarillas A. (2005). Classical theoretical characterization of the surface plasmon absorption band for silver spherical nanoparticles suspended in water and ethylene glycol. Phys. E Low-Dimens. Syst. Nanostructures.

[57] Diniz F.R., Maia R.C.A.P., Rannier L., Andrade L.N., Chaud M.V., da Silva C.F., Corrêa C.B., de Albuquerque Junior R.L.C., da Costa L.P., Shin S.R. (2020). Silver nanoparticles-composing alginate/gelatine hydrogel improves wound healing in vivo. Nanomaterials.

[58] Slistan-Grijalva A., Herrera-Urbina R., Rivas-Silva J.F., Ávalos-Borja M., Castillón-Barraza F.F., Posada-Amarillas A. (2005). Assessment of growth of silver nanoparticles synthesized from an ethylene glycol-silver nitrate-polyvinylpyrrolidone solution. Phys. E Low-Dimens. Syst. Nanostructures.

[59] Stojkovska J., Kostić D., Jovanović Ž., Vukašinović-Sekulić M., Mišković-Stanković V., Obradović B. (2014). A comprehensive approach to in vitro functional evaluation of Ag/alginate nanocomposite hydrogels. Carbohydr. Polym..

[60] Surudžić R., Janković A., Bibić N., Vukašinović-Sekulić M., Perić-Grujić A., Mišković-Stanković V., Park S.J., Rhee K.Y. (2016). Physico-chemical and mechanical properties and antibacterial activity of silver/poly(vinyl alcohol)/graphene nanocomposites obtained by electrochemical method. Compos. Part B Eng..

[61] Badita C.R., Aranghel D., Burducea C., Mereuta P. (2020). Characterization of sodium alginate based films. Rom. J. Phys..

[62] Djošić M., Janković A., Stevanović M., Stojanović J., Vukašinović-Sekulić M., Kojić V., Mišković-Stanković V. (2023). Hydroxyapatite/poly(vinyl alcohol)/chitosan coating with gentamicin for orthopedic implants. Mater. Chem. Phys..

[63] Muangsri R., Chuysinuan P., Thanyacharoen T., Techasakul S., Sukhavattanakul P., Ummartyotin S. (2022). Utilization of freeze thaw process for polyvinyl alcohol/sodium alginate (PVA/SA) hydrogel composite. J. Met. Mater. Miner..

[64] Ghorbel N., Kallel A., Boufi S. (2019). Molecular dynamics of poly(vinyl alcohol)/cellulose nanofibrils nanocomposites highlighted by dielectric relaxation spectroscopy. Compos. Part A Appl. Sci. Manuf..

[65] Wu Y., Yang Y., Zhang Z., Wang Z., Zhao Y., Lei S. (2018). A facile method to prepare size-tunable silver nanoparticles and its antibacterial mechanism. Adv. Powder Technol..

[66] Bondarenko O., Sihtmäe M., Kuzmičiova J., Ragelienė L., Kahru A., Daugelavičius R. (2018). Plasma membrane is the target of rapid antibacterial action of silver nanoparticles in Escherichia coli and Pseudomonas aeruginosa. Int. J. Nanomed..

[67] Rajeshkumar S., Bharath L.V., Geetha R., Shukla A.K., Iravani S. (2019). Broad spectrum antibacterial silver nanoparticle green synthesis: Characterization, and mechanism of action. Micro and Nano Technologies, Green Synthesis, Characterization and Applications of Nanoparticles.

[68] Sjögren G., Sletten G., Dahl E.J. (2000). Cytotoxicity of dental alloys, metals, and ceramics assessed by Millipore filter, agar overlay, and MTT tests. J. Prosthet. Dent..

[69] Phillips H.J., Kruse P., Patterson M. (1973). Dye Exclusion Tests for Cell Viability. Tissue Culture: Methods and Applications.

[70] Rescigno A. (2003). Foundations of Pharmacokinetics.

[71] Mišković-Stanković V.A.T., Mišković-Stanković V., Atanackovic T. (2024). Novel Antibacterial Biomaterials for Medical Applications and Modeling of Drug Release Process.

